# Extra-Limites

**Published:** 1837-10-01

**Authors:** 


### EXTRA-LIMITES.

### THE RAILROAD STEAMER.

By JAMES JOHNSON, M.D.

Were any of the ancients to rise from their tombs, and to behold a steam-ship, full of passengers, darting up the Thames, or a train of carriages, with a thousand people, flying along a railroad, at the rate of 30 miles an hour, they would be very apt to doubt the fact of their revisit to the same planet they had left—since a thousand years in the grave may probably seem no longer than a short siesta after dinner. Their surprise would not be much lessened by the sight of a column of brilliant flame springing up from the middle of a street, or issuing from ten thousand metallic tubes, and turning the darkness of night into the glare of day! If, while gazing at these phenomena, they saw a man, or even a monkey, descend from the clouds suspended as the pendulum of a huge umbrella, they would no longer doubt that they had got into "another if not a better world" than that of their birth and death!

But to return to the Railroad Steamer. Without rudder or rein—without tug or tow-rope—without chart or compass—without impulse from man, or traction from beast—this maximum of power in minimum of space—this magic automaton, darts forward, on iron pinions, like an arrow from a bow, along its destined course. Devised by science, but devoted to industry—harmless as the dove, if unopposed; but fatal as the thunderbolt, if obstructed in its career—this astonishing offspring of human invention—this giant in strength, though a dwarf in stature, drags along, and apparently without effort, whole cargoes of commerce—merchants and their merchandize, artizans and their arts, travellers and their traffic, tourists and their tours (some of them *heavy* enough)—in short, every thing that can be chained to the tail of this Herculean velocipede!

The steam-carriage nearly annihilates distance between the inhabitants of a state, and thereby converts, as it were, a whole country into a city, securing all the good effects of combination and concentration, without the detrimental consequences of a crowded population. By the rail-road, Liverpool, Manchester, Birmingham, and the Metropolis, are constituted *contiguous* cities, while wide and fertile tracts of country intervene! Thus steam multiplies the products of human labour, by increasing their sale and diminishing their price. It will enable us to convert millions of acres from pasturage into corn-fields, and consequently the provender of horses into food for man.

The whole transit of a railroad steamer is a series of miracles, which, in former days, would have been attributed to angels or demons. At starting, the mighty automaton suddenly suppresses his torrent of hissing steam, and belches forth a deep and hollow cough, which is reiterated at shorter and shorter periods, like a huge animal panting for breath, as the engine, with its train, labours up the ascent from Euston-square. These belchings more nearly resemble the pantings of a lion or tiger than any other sound that I know of. With the slow motion, on any considerable ascent, the breathing of the animated machine appears to become more laborious, and the explosions more distinct, till at length the animal seems exhausted, and groans, as it were, under the tremendous effort. But the engine, having mastered the difficulty, acquires velocity before it plunges into the dark abyss of the tunnel under Primrose-hill. There the peal of thunder—the sudden immersion in cimmerian darkness—the clash of reverberated sounds in confined space—the atmospheric chill that rushes over the frame—all combine to induce a momentary shudder at the thought of some possible collision or catastrophe in this subterranean transit, which is increased rather than diminished by the gleams of dubious light that occasionally break in from above, or the sparks of fire that issue every instant from the chimney, rendering "darkness visible." On emerging from the gloomy and gelid cavern, every thing appears of dazzling brightness, and we breathe with delight the pure atmosphere of Heaven.

The moment the highest point of elevation on any part of the road is gained, and a descent commences, the engine, with its long train, starts off with augmenting velocity, dashing along, like lightning, and with an uniform growl or roar, like a continuous discharge of distant artillery or thunder. The scene is now grand—I had almost said terrific. Although it may be a complete calm, the wind appears like a hurricane; and, while the train is flying along the raised embankments, as near Watford, it is impossible not to feel some sense of danger, or an apprehension that some unexpected impediment may hurl the whole cavalcade into the yawning gulf below!

The meetings of the trains flying in opposite directions are scarcely less agitating to the nerves than the transits through the tunnels. The velocity of their course—the propinquity, or apparent identity of the iron trajets along which these hissing meteors move, raise the involuntary but frightful thought of a possible collision, with all its horrible consequences! The period of suspense, however, is but momentary. An electrifying concussion, as it were, of sense, sight, and sound takes place, and, in a few seconds, the object of terror is out of view behind.

But such Herculean labour cannot be carried on in so small a compass, without great expenditure. The AUTOMATON thirsts—he knows the places of refreshment—utters a loud and piercing whistle or note of preparation—slackens his pace—halts at the fountain, and ingurgitates a deluge of water to quench his burning drought. In five minutes he is able to renew his gigantic task!

The steam-shriek is a new phenomenon on the railroad, and a very startling one it is. By opening a small valve in the boiler, a volume of steam is driven, with tremendous force and velocity, through a narrow aperture, in imitation of a throat, causing a shrill shriek, unlike the voice of man, or of any known animal, but so loud as to be heard two miles off. It is a most unearthly yell, or scream, or whistle, which was compared by a distinguished poet, who sat beside me,* to the cry of some monstrous animal while being gored to death. It forms an excellent alarum, to clear the road for the train, and apprize those at the stations, that the engine approaches.

The rail-road travelling possesses many peculiarities, as well as advantages, over the common modes of conveyance. The velocity with which the train moves through the air is very refreshing, even in the hottest weather, where the run is for some miles. The vibratory, or rather oscillatory motion communicated to the human frame, is very different from the swinging and jolting motions of the stage coach, and is productive of more salutary effects. It equalizes the circulation, promotes digestion, tranquillizes the nerves (after the open country is gained), and often causes sound sleep during the succeeding night, the exercise of this kind of travelling being unaccompanied by that lassitude, aching, and fatigue which, in weakly constitutions, prevents the nightly repose. The railroad bids fair to be a powerful remedial agent in many ailments to which the metropolitan and civic inhabitants are subject.

To those who are curious, and not very timid, the open carriages are far preferable to the closed ones, especially in fine weather. In bad weather, and particularly at first, invalids may travel with more advantage under cover. I have no doubt that to thousands and tens of thousands of valetudinarians in this overgrown Babylon, the run to Boxmoor or Tring and back, twice or thrice a week, will prove a means of preserving health and prolonging life, more powerful than all the drugs in Apothecaries' Hall.

In fine, a man may travel from the pole to the equator—

"A Gadibus usque ad Gangem"—

without seeing any thing half so astonishing as the wonders of the railroad. The pangs of Etna, and the convulsions of the elements excite feelings of horror and terror, without any thing of pride. The Magic—the miracles of the railroad engender an exulting consciousness of superiority in the genius of man, more intense and conclusive than any effort of poet, painter, or philosopher.

The railroad journey, however, is not without its inconveniences, many of

---

* Campbell.

which may be prevented by a little ingenuity. The greatest is the discharge of cinders, some of them ignited, from the chimney, which are not only disagreeable but occasionally dangerous to the eyes of those in the open carriages. This might be prevented by an awning—a protection which is adopted on some railroads, and one that must ultimately be adopted on all. It is a protection from the elements of fire and water which every company is bound to afford to the passengers, and is attended with trifling expense. Till then, glasses or a veil are necessary guards for the eyes.

The transits of the tunnels, in hot weather, causing a sudden vicissitude of temperature, to the extent of 20 degrees of the thermometer, or thereabouts require some precaution on the part of sensitive invalids. A shawl or large handkerchief, thrown over the head, is a sufficient protection, and those who do not take this measure, should keep their eyes shut, during the passage, since sparks and cinders are, unavoidably, thrown in closer showers over the passengers here than in the open space.

To speculate on the moral, physical, political, and economical effects and consequences of railroads and steam navigation, when carried to their full extent, is beyond my province—perhaps beyond the bounds of human foresight. If the semi-civilized peasants of the remotest isles of the Hebrides, of Orkney, and of Shetland, can, even now, transmit, in a few hours, the produce of their huts, their mountains, their moors, and their farm-yards, to the markets of Glasgow and Edinburgh, so as, in three or four days, to pay the annual rents of their tenements and wildernesses, what may we not expect from the extension and perfection of this facility of intercommunication? In days of yore, the imponderable products of the intellect travelled as slowly as the material merchandize of mankind. They will now be diffused, from the centre to the periphery—from the remotest outlines to the foci of society, with a rapidity little less than that of *thought* itself!! The ultimate *consequences* cannot be appreciated at present; but we may safely conclude that the benevolent Author of our existence did not endow the mind of MAN with such extraordinary powers of invention, without the DESIGN of final advantage to his physical wants, his social relations, and his spiritual nature.

